# Kyste hydatique rénal fistulisé dans les voies urinaires chez un hémodialysé chronique

**DOI:** 10.11604/pamj.2017.28.219.13790

**Published:** 2017-11-09

**Authors:** Nadia Kabbali, Tarik Sqalli

**Affiliations:** 1 Service de Néphrologie, CHU Hassan II, Fès, Maroc; 2 Equipe de Recherche REIN, Faculté de Médecine et de Pharmacie, Université Sidi Mohamed Ben Abdellah, Fès, Maroc

**Keywords:** Kyste hydatique rénal, hémodialyse, Maroc

## Abstract

Hydatidosis is a parasitic infection caused by the larval stage of Echinococcus granulosus. Renal hydatic cyst is unusual. It may fistulize into the urinary excretory system, requiring specific management. The aim of our study was to highlight the diagnostic and therapeutic specificity of this pathology in patients on chronic hemodialysis among whom diuresis is not always preserved, in order to focus attention on hydaturia and to give an indication to diagnosis. We report the case of a 47-year old patient with a 4-year history of hemodialysis due to glomerular nephropathy detected at the final stage. Moreover, the patient had a 6-month histoiry of impaired general condition associated with right lower back pain, without fever. Abdominal CT scan showed right kidney measuring 13.4cm, with important ureteropyelocaliceal dilation, pushing the renal parenchyma associated with renal pelvis measuring 4.3cm, without detectable lithiasic obstacle. However, it showed membranous cyst at the level of the lower right renal polar parenchyma, measuring approximately 76.5 x 54 mm, contacting the renal pelvis, also containing a few membranes. The patient underwent thorough interview revealing the presence of hydaturia in the few drops of residual diuresis. Given that the patient had end stage chronic renal failure and that he was under renal replacement therapy, therapeutic approach was based on total nephrectomy.

## Image en médecine

L'hydatidose est une parasitose liée au développement de la forme larvaire d'Echinococcus granulosus. Le kyste hydatique rénal constitue une localisation inhabituelle. Il peut se fistuliser dans les voies excrétrices urinaires et nécessiter alors une prise en charge spécifique. Nous présentons ici un cas témoignant de la spécificité diagnostique et thérapeutique de cette pathologie chez les patients en hémodialyse chronique, chez qui la diurèse n’est pas toujours conservée pour témoigner de l’hydaturie et orienter le diagnostic. Il s’agit donc d’un patient de 47 ans, en hémodialyse depuis 4 ans pour une néphropathie glomérulaire découverte au stade terminal. Le patient présente depuis 6 mois une altération de l’état général avec des lombagies droites, sans fièvre. La TDM abdominale montre un rein droit mesurant 13,4cm, siège d’une importante dilatation urétéro-pyélo-calicielle laminant le parenchyme rénal, avec un pyélon mesuré à 4,3cm, sans obstacle lithiasique visible. Cependant on individualise au niveau polaire inférieur droit, une formation kystique contenant des membranes, mesurant approximativement 76,5 x 54mm, communiquant avec le pyélon qui contient également quelques membranes. Un interrogatoire plus laborieux a révélé la présence d’une hydaturie sur les quelques gouttes de diurèse résiduelle. Vu que le patient est en insuffisance rénale chronique terminale en traitement de suppléance, le choix thérapeutique s’est porté sur une néphrectomie totale.

**Figure 1:**
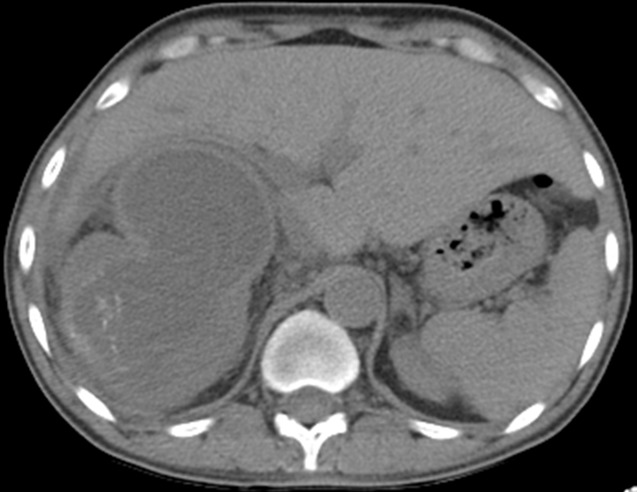
Image scannographique en rapport avec un kyste hydatique rénal polaire inférieur droit, fistulisé dans les voies urinaires droites, compliqué d’une importante hydronéphrose, chez un patient en hémodialyse chronique

